# Basal Heat Capacity of Skinned Skeletal Muscle with Selective Removal and Denaturation of Myoproteins: A Study with Differential Scanning Calorimetry

**DOI:** 10.3390/ijms27020710

**Published:** 2026-01-10

**Authors:** Naoya Nakahara, Tetsuo Ohno, Sumiko Kimura, Maki Yamaguchi, Shigeru Takemori

**Affiliations:** 1 Department of Molecular Physiology, The Jikei University School of Medicine, Tokyo 105-8461, Japanmaki@jikei.ac.jp (M.Y.); sml@jikei.ac.jp (S.T.); 2 Faculty of Health Care and Medical Sports, Teikyo Heisei University, Chiba 290-0193, Japan; t.ohno@thu.ac.jp

**Keywords:** heat capacity, differential scanning calorimetry, actin, myosin

## Abstract

The specific heat capacity of skinned muscle in an adhering rigor solution was studied with differential scanning calorimetry (DSC) heating runs to search for a heat sink in the sarcomere of the muscle. To elucidate the contribution of major myoproteins to heat capacity, myosin and actin were partially removed by high-KCl and gelsolin treatments, respectively. Differential heat denaturation of myosin (together with α-actinin) and actin was induced to confirm their contributions. On the DSC curve, aside from the endothermic peaks representing ice melting and protein denaturation, the steady baseline level showed a significant increase in basal heat capacity in the presence of skinned muscle compared to the rigor solution alone. In the physiological temperature range from 10 to 25 °C, untreated skinned muscle in the native state (non-denatured) introduced an extra basal heat capacity of 0.4 J K^−1^ (g evaporable weight)^−1^, which was diminished by both removing and denaturing actin and was additionally increased by removing myosin; myosin denaturation had little effect on the basal heat capacity. Based on these results, we considered actin to be the fundamental source of extra basal heat capacity, which was partly suppressed by the thermally stable region of myosin under rigor conditions. This extra basal heat capacity was roughly preserved at sub-zero temperatures, suggesting the involvement of non-freezing water molecules. The extra basal heat capacity may have contributed to thermal buffering during muscle function via actin-associated hydration. As a supplemental result, we found a small reversible endothermic peak around −21 °C, which was suppressed in the presence of skinned muscle. Heating beyond the denaturing temperatures reduced this suppression effect.

## 1. Introduction

Myosin and actin are the major myoproteins in skeletal muscle sarcomeres. The head of myosin, which protrudes from the thick filament backbone, cleaves MgATP to fuel contractile interactions with actin along the thin filament [1,2]. The limited volume of water molecules and solutes filling the interfilament space of the lattice of thick and thin filaments is expected to serve as a significant heat sink in the sarcomere. Previous biophysical studies have demonstrated that water surrounding filamentous actin exhibits properties distinct from bulk water, including enhanced mobility, often referred to as “hyper-mobile” [3,4,5]. These unique hydration properties suggest that actin-associated water may significantly contribute to muscle heat capacity, rather than merely serving as a passive solvent.

Nuclear magnetic resonance (NMR) studies using skinned (demembranated) muscle fibers have demonstrated that the properties and distribution of water in muscles depend on the structural and biochemical state of the sarcomere, indicating that protein–water interactions are dynamically regulated in situ [6,7,8]. However, in principle, NMR measurements are unable to quantitatively assign the properties of distinct water populations to myoproteins.

Differential scanning calorimetry (DSC) offers a thermodynamic approach for characterizing water states. Unlike spectroscopic techniques, DSC can not only capture discrete thermal transitions but also continuous changes in the baseline heat capacity with temperature, which reflects the collective behavior of water molecules and their interactions with surrounding proteins. Such baseline heat capacity contains information on the thermal degrees of freedom of the muscle system and may therefore provide insight into how protein–water interactions contribute to thermal buffering in the muscle as a heat sink.

Previous DSC studies on glycerinated or skinned muscle fibers have provided valuable insights into the thermal stabilities of myosin, actin, and associated proteins under different biochemical states, including intermediate states of ATP hydrolysis cycle [9,10]. These studies primarily analyzed discrete thermal transitions and denaturation enthalpies to characterize structural changes in contractile proteins. However, the changes in the thermodynamic properties of water arising from its interactions with myoproteins have received little attention. Aubin et al. reported that intact muscle tissue had an additional melting peak at −5 °C to −10 °C and attributed this to intermediate water states, suggesting a substantial contribution from protein-associated water [11].

Changes in the heat capacity of purified actin upon polymerization have been reported by Quirion and Gicquaud [12], who demonstrated that actin polymerization contributes to its intrinsic thermodynamic properties. However, that study was performed on isolated actin at a fixed temperature and did not address the temperature-dependent baseline heat capacity of skeletal muscles as an integrated sarcomeric system. Although studies using purified or reconstituted proteins have provided valuable insights into intrinsic protein properties, they lack the highly ordered sarcomeric architecture and restricted interfilament space present in the muscle.

Because the basal heat capacity is largely governed by interactions between macromolecules and the surrounding water, its analysis may provide insights into how sarcomeric proteins modulate intracellular water thermal properties. Therefore, identifying the molecular origin of this basal heat capacity is crucial for understanding the physical basis of heat buffering in muscles.

In this study, DSC was performed using skinned muscle fibers to study the specific heat capacity of skeletal muscle, because these fibers retain this native structural organization while permitting controlled manipulation of biochemical conditions, making them particularly suitable for analyzing protein–water interactions relevant to muscle heat capacity. By analyzing the DSC baseline level over both physiological and sub-zero temperature ranges, we aimed to clarify the role of myoprotein–water interactions as potential heat sinks in sarcomeres.

The use of skinned muscle allowed the natural myoplasmic solution containing soluble non-sarcomeric proteins to be replaced with an artificial salt solution. The source of the heat capacity was distinguished by the selective removal of myosin and actin using high-KCl and gelsolin treatments, respectively; step-wise thermal denaturation of myosin and actin; and freezing/melting of the aqueous solution component.

## 2. Results

For clarity, the experimental states and terminology used throughout the Results section are defined below.

All measurements were performed using skinned (demembranated) skeletal muscle fibers, in which the sarcolemmal membrane system was removed while preserving the sarcomeric structure and function. “Untreated skinned muscle” refers to skinned muscle fibers without selective protein extraction, while “high-KCl-treated muscle” and “gelsolin-treated muscle” refer to skinned muscle fibers in which myosin and actin were preferentially removed, respectively.

The term “native state” is used to describe the initial condition of skinned muscle specimens prior to any heating beyond physiological temperature, i.e., before the irreversible thermal denaturation of myoproteins occurred. In contrast, the terms “after heating to 60 °C” and “after heating to 80 °C” refer to specimens that had undergone irreversible denaturation of myosin (together with α-actinin) and actin, respectively, as confirmed by electron microscopy and DSC profiles.

Throughout this experiment, comparisons are made between these defined experimental states, and all quantitative analyses of DSC curves are based on heating scans.

### 2.1. Selective Removal of Myoproteins

**Figure 1:**
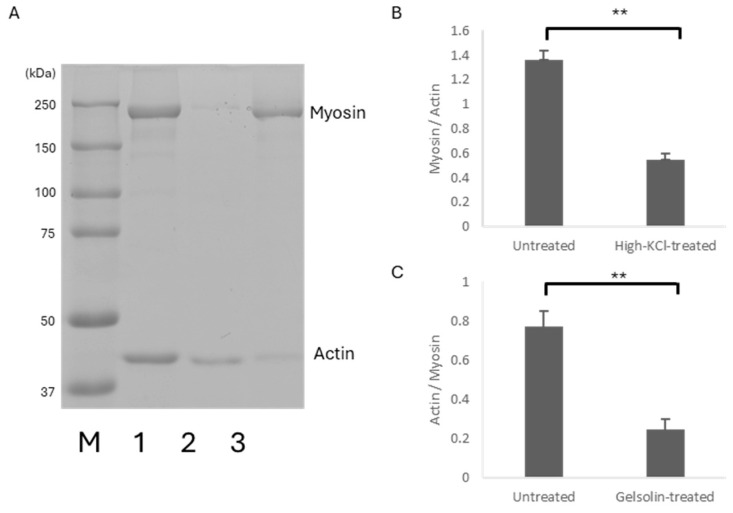
(**A**): Coomassie Brilliant Blue-stained 8% SDS-PAGE image of the untreated (1), high-KCl-treated (2), and gelsolin-treated (3) skinned muscle used for DSC analysis. Protein molecular weight markers (kDa) (M) are shown on the left. (**B**): The ratio of the myosin heavy chain band density relative to actin in the untreated (*n* = 15) and high-KCl-treated skinned muscle (*n* = 28). (**C**): The ratio of the actin band density relative to myosin heavy chains in the untreated (*n* = 15) and gelsolin-treated skinned muscle (*n* = 15). Error bars indicate SEM. ** *p* < 0.01.

Electrophoretic analysis (Figure 1) showed that the band density of myosin relative to actin decreased to 40% in high-KCl-treated muscle compared to untreated skinned muscle (Figure 1B). In contrast, the band density of actin relative to myosin in gelsolin-treated muscle decreased to 32% of that in untreated skinned muscle (Figure 1C). These results indicated that high-KCl and gelsolin treatments preferentially reduced the amount of myosin and actin in the muscle, respectively.

### 2.2. Step-Wise Denaturation of Myoproteins

**Figure 2:**
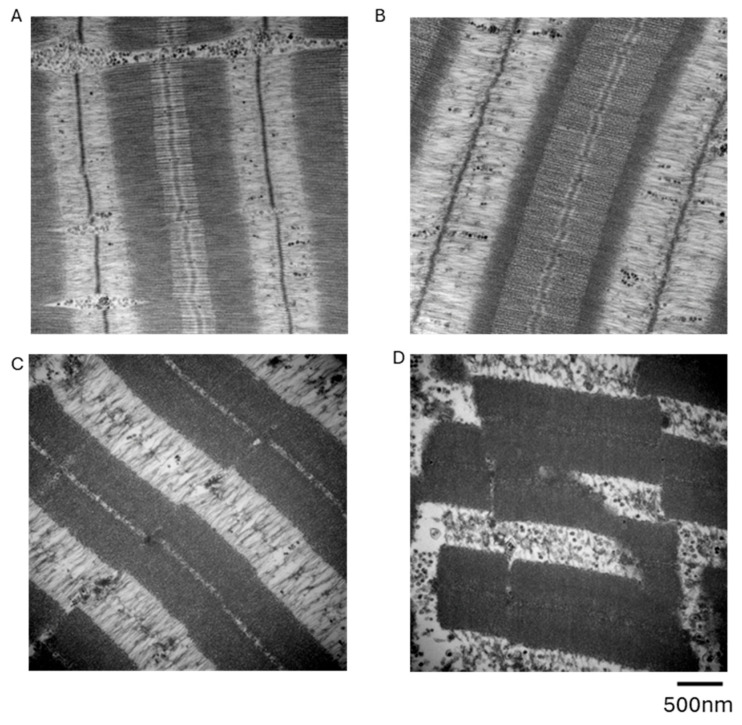
Electron micrographs of the muscle specimen. Before freezing (**A**) and after the initial heating run from −80 °C to 30 °C (**B**), to 60 °C (**C**), and to 80 °C (**D**).

We compared the electron micrographs of the untreated skinned muscle at various steps of heating/cooling DSC runs performed at a rate of 1 °Cmin^−1^. When skinned muscle, cooled to −80 °C, was subsequently heated up to 30 °C, the fine sarcomere structure was largely preserved (Figure 2A,B). When heated up to 60 °C, beyond the probable denaturation temperature of myosin [10] and α-actinin (main component of Z-line) [13], the A-band and Z-line of the sarcomere were selectively deformed (Figure 2C). When heated up to 80 °C, beyond the reported denaturation temperature of actin [10], the overall sarcomere integrity was destroyed (Figure 2D), likely as a consequence of myosin, α-actinin, and actin denaturation.

### 2.3. DSC Curves

**Figure 3:**
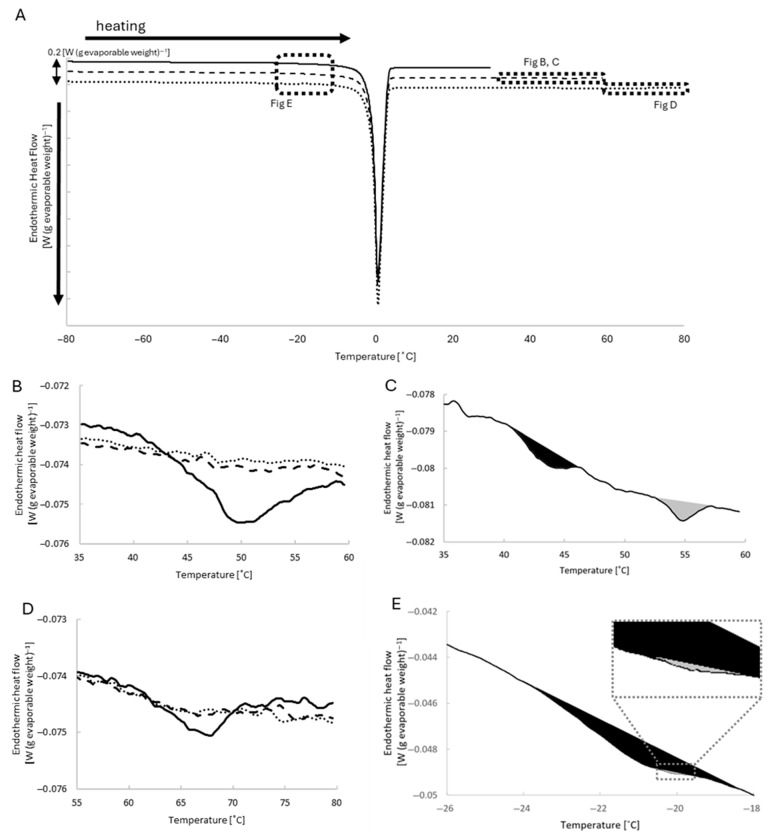
(**A**): Representative DSC curves obtained from the untreated skinned muscle during the initial heating runs from −80 °C to 30 °C (solid), 60 °C (dashed), and 80 °C (dotted). The curves have been shifted vertically for clarity. (**B**): The peak at 50 °C in the first (solid), second (dashed), and third (dotted) runs up to 60 °C of the untreated muscle. Note the broad base of the peak. (**C**): The peaks around 50 °C in the first run up to 60 °C of the muscle pretreated with high-KCl solution. Note the small separate peaks at 44 °C (black) and 55 °C (gray). (**D**): The peak at 67 °C in the first (solid), second (dashed), and third (dotted) runs up to 80 °C of the untreated muscle. (**E**): The peak at −21 °C (black) during the initial heating run up to 30 °C of the untreated muscle. Note a small peak at −20 °C (gray).

The representative DSC curves of heating runs from −80 °C up to 30 °C, 60 °C, and 80 °C are shown in Figure 3A.

Heating scans were analyzed without subtracting the rescan baseline to not only evaluate discrete endothermic transitions but also the steady baseline level of the DSC curves, the latter of which refers to the temperature-dependent heat flow outside distinct endothermic peaks, reflecting the apparent specific heat capacity of the specimen.

In addition to the large endothermic peak associated with ice melting around 0 °C, smaller endothermic peaks were reproducibly observed at approximately −21 °C, 50 °C, and 67 °C in the initial heating scans of skinned muscles. The peaks at higher temperatures disappeared in the subsequent heating runs, indicating irreversible thermal denaturation of specific myoproteins. The peak around −21 °C, however, was retained even after heating to 80 °C.

#### 2.3.1. Peaks Around 50 and 67 °C

During the first heating runs up to 60 °C (beyond the denaturation temperature of myosin [10] and α-actinin [13]) of untreated muscle, there was a broad peak at 50 °C. This peak was not observed in subsequent runs (Figure 3B). High-KCl treatment to remove myosin significantly diminished the main peak at 50 °C, leaving small distinct peaks at 44 and 55 °C (Figure 3C, Table 1).

A peak at 67 °C was observed during the first heating runs up to 80 °C (beyond the denaturation temperature of actin [10]), and was not observed in subsequent runs (Figure 3D). Treatment with gelsolin to remove actin significantly reduced the peak area (Table 2).

#### 2.3.2. Peaks Around −21 °C

**Figure 4:**
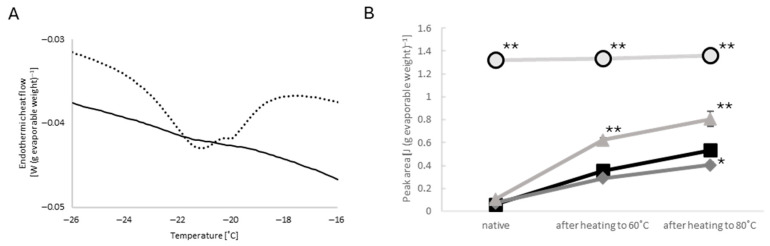
(**A**): A typical trace of the −21 °C peak of the rigor solution alone (dotted) and of the untreated muscle in the native state (solid). (**B**): Averaged area of the −21 °C peak of the rigor solution alone (light gray circles with black outline, *n* = 4 specimens), untreated muscle (black square, *n* = 7 specimens), high-KCl-treated muscle (deep gray diamond, *n* = 27 specimens), and gelsolin-treated muscle (light gray triangle, *n* = 4 specimens) in the native state after heating to 60 °C and to 80 °C. Error bars indicate SEM, if visible. ** *p* < 0.01; * *p* < 0.05 vs. untreated.

A small peak was found at −21 °C with a small sub-peak at −20 °C in all the heating runs of muscle specimens (Figure 3E). Surprisingly, the heating scan of the rigor solution without the skinned muscle showed a larger peak at the corresponding temperature. The area of the peak observed with rigor solution alone was 1.33 ± 0.03 J (g evaporable weight)^−1^ (*n* = 4 specimens), which was 22 times larger than the peak area observed in the presence of untreated skinned muscle in the native state (Figure 4). Heating scans of rigor solution alone up to 80 °C had no effect on the peaks (Figure 4B), and the presence of skinned muscle suppressed the peak at −21 °C.

The high-KCl and gelsolin treatments had no appreciable effect on the suppression of the −21 °C peak of rigor solution before heating beyond 30 °C (Figure 4B). The suppressing effect was found to be significantly reduced with a rise in the heating temperature range (up to 60 and up to 80 °C). The reduction in the suppressive effect by heating was slightly smaller in the high-KCl-treated muscle, but significantly larger in the gelsolin-treated muscle (Figure 4B). The −20 °C sub-peak behaved similarly to the −21 °C peak, with a minor difference being that heating from 60 °C to 80 °C produced no appreciable change in the −20 °C sub-peak area in the gelsolin-treated muscle.

#### 2.3.3. Peak Around 0 °C

**Figure 5:**
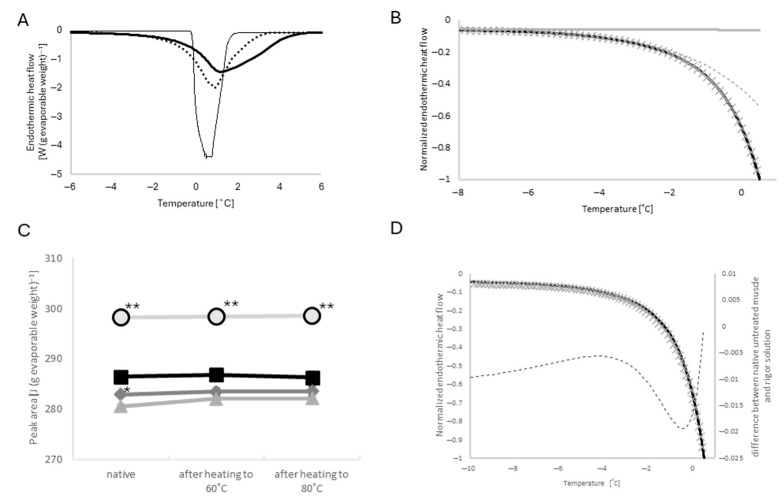
(**A**): The peak around 0 °C of pure water (thin solid), the rigor solution alone (dotted), and the untreated muscle in the native state (thick solid). (**B**): DSC curve of the untreated skinned muscle in the native state deviating from the baseline toward the 0 °C peak (black solid) could be cumulatively fitted with linear (gray solid), gentle exponential (gray dashed), and steep exponential terms (gray cross mark). (**C**): Averaged area of the 0 °C peak of the rigor solution alone (light gray circles with black outline, *n* = 4 specimens) and the untreated (black square, *n* = 7 specimens), high-KCl-treated (deep gray diamond, *n* = 27 specimens), and gelsolin-treated muscle (light gray triangle, *n* = 4 specimens) in the native state after heating to 60 °C and 80 °C. Error bars indicate SEM, if visible. * *p* < 0.05; ** *p* < 0.01 vs. untreated. Heating had no statistically significant effect between any group. (**D**): Heat flow of the rigor solution alone (black solid; left axis) and the untreated muscle normalized for a 4% decrease in the peak area (gray cross mark; left axis) and their difference (gray dashed; right axis).

At the melting temperature of ice, around 0 °C, each rigor solution and skinned muscle specimen showed a corresponding peak (Figure 5A). Unlike the single exponential development of the pure water 0 °C peak, the peaks of rigor solution and skinned muscle specimens were multi-exponentially broader and showed depression of the melting point by more than 0.8 °C.

When compared with rigor solution alone, the presence of skinned muscle caused a 4% decrease in the peak area and heating up to 80 °C, and high-KCl/gelsolin pre-treatment had no further effect on the area (Figure 5C). When normalized for the 4% difference, the skinned muscle peak showed only a minor difference from that of the rigor solution alone (Figure 5D), peaking at −0.5 °C.

#### 2.3.4. Steady Baseline Level

**Figure 6:**
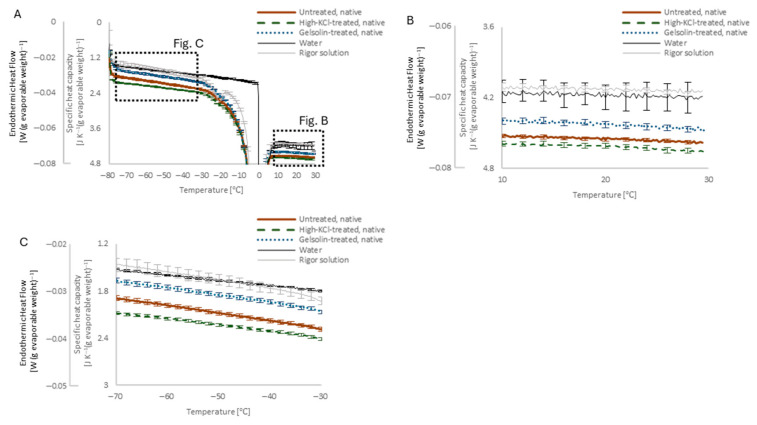
The steady baseline level of the averaged DSC curves of the skinned muscle in the native state. From the proportionality at the steady baseline level, the specific heat capacity is shown as the second vertical axis. Steady DSC baseline levels of the untreated (*n* = 7 specimens), high-KCl-treated (*n* = 27 specimens), and gelsolin-treated (*n* = 4 specimens) muscle in the native state (**A**) are shown separately for the physiological temperature range (**B**) and sub-zero temperature range (**C**) with the baseline levels of pure water (*n* = 3 specimens) and rigor solution (*n* = 4 specimens). For clarity, SEM error bars are shown at 2 °C intervals.

The steady baseline level of the DSC curve, defined as the heat flow below and above the melting or denaturing peak, where no distinct endothermic transitions were observed, was analyzed to evaluate the basal heat capacity. The steady baseline level of the rigor solution was almost identical to that of pure water (Figure 6), and the presence of the skinned muscle decreased the baseline level below and above the 0 °C peak, indicating an increase in the basal heat capacity (Figure 6B,C). At the temperature range from 10 to 25 °C, the increase in the specific heat capacity was found to be 0.4, 0.5, and 0.3 JK^−1^ (g evaporable weight)^−1^ in the untreated, high-KCl-treated, and gelsolin-treated skinned muscle, respectively (Figure 6B).

**Figure 7:**
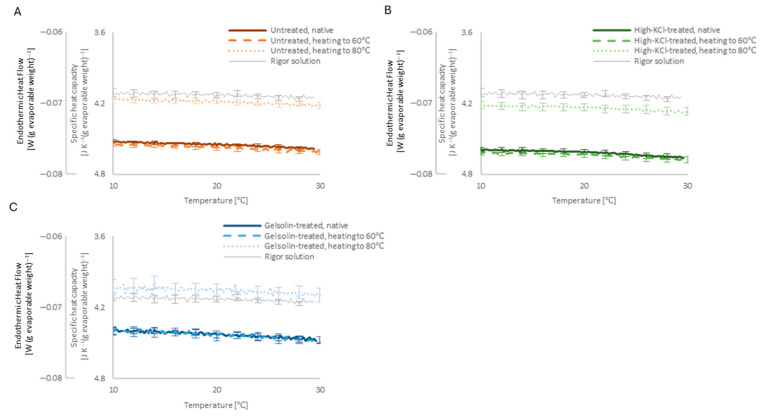
The steady baseline of the averaged DSC curves in the native state after heating to 60 °C and 80 °C of the untreated ((**A**); *n* = 7 specimens), high-KCl-treated ((**B**); *n* = 27 specimens), and gelsolin-treated ((**C**); *n* = 4 specimens) skinned muscle at the physiological temperature range. From the proportionality at the steady baseline level, the specific heat capacity is shown as the second vertical axis. The baseline level of the rigor solution in Figure 6 is shown repeatedly for reference. For clarity, SEM error bars are shown at 2 °C intervals.

Heating up to 60 °C (beyond the myosin denaturation temperature) had no appreciable effect on the DSC baseline level of the untreated and high-KCl/gelsolin-treated muscle (Figure 7). Heating up to 80 °C (beyond the actin denaturation temperature) increased the DSC baseline level of untreated and high-KCl/gelsolin-treated muscles close to that of rigor solution alone.

**Figure 8:**
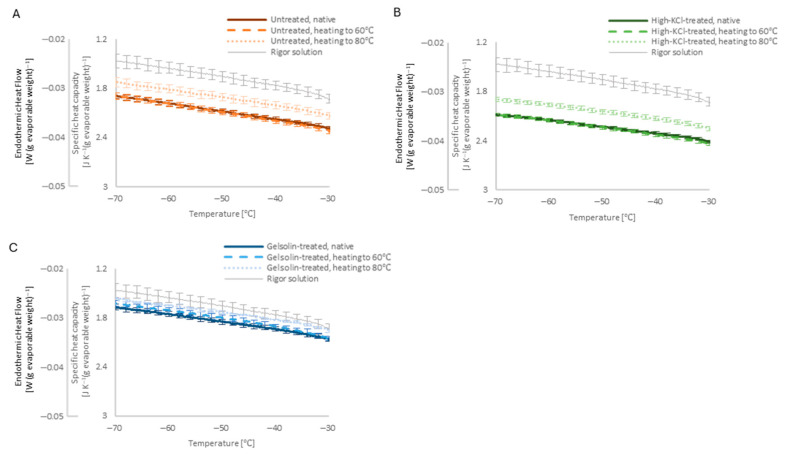
The steady baseline of the averaged DSC curves in the native state after heating to 60 °C and 80 °C of the untreated ((**A**); *n* = 7 specimens), high-KCl-treated ((**B**); *n* = 27 specimens), and gelsolin-treated ((**C**); *n* = 4 specimens) skinned muscle at the sub-zero temperature range. From the proportionality at the steady baseline level, the specific heat capacity is shown as the second vertical axis. The baseline level of the rigor solution in Figure 6 is shown repeatedly for reference. For clarity, SEM error bars are shown at 2 °C intervals.

The decrease in the DSC baseline level from that of the rigor solution alone was also observed at temperatures well below 0 °C at an extent roughly consistent with physiological temperature for the untreated and high-KCl-treated skinned muscle in the native state (Figure 8), but the effect of heating up to 80 °C (beyond the actin denaturation temperature) had a relatively smaller effect.

## 3. Discussion

In this study, we investigated the molecular origin of basal heat capacity of skeletal muscle using the DSC of skinned muscle fibers with selective removal and thermal denaturation of major myoproteins. Our results demonstrated that actin, rather than myosin, is the principal contributor to the extra basal heat capacity of sarcomeres in the physiological temperature range.

### 3.1. Denaturation of Myosin and Actin

Skinned muscle specimens showed large peaks at 50 and 67 °C (Figure 3B,D) only in the first heating scan beyond each temperature. Selective removal of myosin and actin (Table 1 and Table 2) and electron microscopic observation (Figure 2) confirmed that the 50 and 67 °C peaks mainly represented irreversible heat denaturation of myosin and actin, respectively.

From the electron microscopic observations, heating up to 60 °C seemed to cause heat denaturation of α-actinin (Figure 2C), which is the main component of the Z-line structure. The α-actinin content in sarcomere was considered to be 1/7 of the myosin content [14]; therefore, it is reasonable to assume that the heat denaturation of α-actinin causes minor contributions to the large myosin denaturation peak at 50 °C (Figure 3B).

The broadness of the 50 °C peak could represent the successive denaturation of the parts of myosin molecules in the highly packed sarcomere structure. A 60% reduction in the myosin content with the high-KCl treatment (Figure 1B) caused splitting of the 50 °C peak to 44 and 55 °C peaks with a reduction in their sum areas to 17% of the original 50 °C peak (Figure 3B,C, Table 1). The observed splitting of the 50 °C peak may also have involved distinct components of myosin or other myoproteins with differential thermal stability [10].

The gelsolin treatment simply reduced the area of the relatively sharp peak at 67 °C to 30% (Table 2), which is consistent with the compact globular conformation of actin and its removal ratio (68%) (Figure 1C).

### 3.2. Steady Baseline Level

The steady baseline level of rigor solution alone at a temperature range from 10 to 25 °C indicated the specific heat capacity of rigor solution to be 4.2 JK^−1^ (g evaporable weight)^−1^, which was almost identical to the basal heat capacity of pure water (Figure 6B). However, in the presence of skinned muscle, the steady baseline levels of the DSC curves dropped significantly from those of the rigor solution alone on an evaporable weight basis, indicating that the presence of skinned muscle adds additional (extra) basal heat capacity to the rigor solution. One may argue that non-evaporable components in the skinned muscle (11–16% of the specimen weight) may carry some extra heat capacity by themselves. However, we consider that any heat capacity in skinned muscle works in thermal equilibrium with the surrounding evaporable water molecules; therefore, it is reasonable to formally handle non-evaporable components as subsidiaries of evaporable water molecules.

The increase in heat capacity attributable to the presence of untreated skinned muscle in the native state was markedly reduced, approaching close to the level of rigor solution alone when using gelsolin treatment of the muscle and actin denaturation (Figure 7). This indicates that by driving the surrounding water molecules, actin serves as a significant source of heat sink of the extra basal heat capacity of 0.4 JK^−1^ (g evaporable weight)^−1^ in the sarcomere, and that losing the effect of actin returned the water molecules to their state in the rigor solution alone, even in the presence of denatured actin. The latter rationalizes the handling of non-evaporable components as subsidiaries for interacting evaporable water molecules.

The 68% removal of actin by the gelsolin treatment (Figure 1C) decreased the extra basal heat capacity by as much as 30% (Figure 6B). This probably indicates that the actin molecules cooperatively drive the water molecules close to the saturation level; thus, denaturation by heating up to 80 °C caused a larger decrease in the extra basal heat capacity closer to the level of rigor solution alone at the physiological temperature range.

The high-KCl-treated skinned muscle showed a higher extra basal heat capacity than the untreated muscle (Figure 6B). Myosin in untreated muscle would have suppressed the heat sink capacity of actin through rigor cross-bridge formation, and high-KCl treatment would have released this suppression with the removal of myosin. Lack of suppression release by myosin denaturation with heating up to 60 °C may further suggest thermal stability of the region of myosin molecules responsible for the suppression. We infer that this region is intrinsically stable or is stabilized by interaction with actin through rigor cross-bridge formation with hydrophobic bonding [15].

Based on the above considerations, it is possible that the physiological dissociation of myosin heads from actin also releases suppressive effect on the heat sink capacity of actin. To test this possibility, we preliminarily tested heating DSC runs of skinned muscle in the relaxed state in the relaxing solution containing sufficient MgATP: Binding of MgATP to myosin heads dissociated the heads from actin, thereby relaxing the muscle. From the heating DSC runs from 10 to 25 °C at two different rates of 1 and 5 °Cmin^−1^, we extracted the heat of ATP hydrolysis to be 0.1 Jmin^−1^ (g evaporable weight)^−1^. Its subtraction indicated that the skinned muscle basal specific heat capacity in the relaxing solution was 0.1 JK^−1^ (g evaporable weight)^−1^ higher than that of the skinned muscle in the rigor solution.

Each skinned muscle condition showed extra basal heat capacity, not only in the physiological temperature range from 10 to 25 °C but also in the sub-zero temperature range from −80 to −30 °C. Unlike at the physiological temperature range, the removal of actin with gelsolin treatment had a larger effect, but actin denaturation with heating up to 80 °C had a smaller effect. The cooperative interaction between actin and the surrounding water molecules is postulated above, and the interaction between denatured actin and the surrounding water molecules is modulated by freezing the majority of the water molecules in the specimen. However, the existence of extra basal heat capacity in the sub-zero temperature range is interesting regarding the extra basal heat capacity source, recalling that the high heat capacity of liquid water is generally ascribed to the breaking and reforming of extensive hydrogen bonding among water molecules. At sub-zero temperatures, where the bulk solution component is frozen to form rigid hydrogen bonds between water molecules, the source of the extra heat capacity is relatively limited to the energy of labile hydrogen bonding between the non-freezing water molecules, or some other form of energy. One of the candidates is the kinetic energy of hyper-mobile water around actin filaments, as proposed by Suzuki et al. based on their dielectric dispersion measurements [3,5].

From the 0 °C peak area, we consider that the evaporable weight of the present skinned muscle specimens is composed of about 96% of the bulk solution that freezes near 0 °C, and the remaining 4% of the solution is non-freezing, at least down to −80 °C, which probably includes at least part of the hyper-mobile water component around the actin filaments. The details of the postulated cooperative interactions between water molecules and actin in the native and denatured states will be the subject of our future studies.

Aubin et al. [11] reported a solution component melting at −5 to −10 °C in barnacle intact muscle. However, no distinct peaks were observed in this temperature range in the present experiments (Figure 5), probably due to the use of skinned muscles that lack soluble myoplasmic proteins. Aubin et al. consistently reported that the DSC peak of intermediate water became less apparent with an increase in the water content of their intact muscle fibers. We hypothesized that the minute peak observed at −0.5 °C within the relatively broad 0 °C peak (Figure 5D) reflects the effect of freezing condensation in skinned muscle. Freezing condensation was evident in rigor solution and skinned muscle from the observation of a larger freezing point depression than the calculated value (−0.4 °C), and the multi-exponential development of the 0 °C peak (Figure 5B).

### 3.3. Peak at −21 °C

Interestingly, we found a small peak at −21 °C, which was largest in rigor solution without skinned muscle and almost disappeared in the presence of the skinned muscles. Our preliminary studies on the rigor solution −21 °C peak indicated the following: (1) the −21 °C peak was primarily influenced by anions in the solution; (2) the relative area was constant irrespective of the total volume of the solution; (3) and the rate of cooling and heating had little effect on the peak area except the disappearance of the −20 °C sub-peak upon ultra-rapid freezing with liquid nitrogen. However, we were unable to specify the etiology of these peaks.

### 3.4. The Broader Context and Relation to Other Works

Skeletal muscle contraction is accompanied by characteristic heat absorption and release, reflecting thermodynamic coupling between ATP hydrolysis and force generation; the muscle force generation involves an endothermic process, indicating that muscles must transiently buffer heat during contraction [2,16]. Despite extensive investigations into muscle energetics, the molecular basis of this intrinsic heat buffering capacity within the sarcomere remains incompletely understood.

Most previous DSC studies on muscle fibers and purified myoproteins have focused on discrete endothermic transitions associated with protein denaturation or conformational changes in myosin and actin [9,10]. In contrast, the steady baseline level of DSC curves, which reflects the basal heat capacity of muscles outside such transitions, has received little attention. In the present study, we directly address this gap by demonstrating that the extra basal heat capacity of skeletal muscle in the physiological temperature range is primarily attributable to actin. Previous calorimetric studies of purified actin demonstrated a decrease in heat capacity upon polymerization, reflecting changes in protein hydration and conformational flexibility [12]. However, these studies did not address the steady baseline heat capacity of muscle or the behavior of actin within a sarcomeric lattice.

In the present study, we concluded that actin is the primary molecular contributor to the basal heat capacity of skeletal muscle, which is consistent with independent biophysical evidence indicating that actin creates a unique hydration environment. Dielectric dispersion measurements revealed the presence of so-called “hyper-mobile water” surrounding actin filaments [3], and subsequent studies showed that myosin binding alters the volume and dynamics of this hydration layer [5]. In parallel, NMR studies using skinned muscle fibers demonstrated that the interaction between myosin and actin modulates intracellular water dynamics, indicating that protein–water interactions are dynamically regulated within the sarcomere [6,7,8]. Recent quantitative MRI studies in the dystrophic skeletal muscle of dogs have shown that bicomponent water T_2_ relaxometry and extracellular volume fraction measurement are useful to evaluate disease activity. Moreover, they also showed that change in the water T_2_ is associated with cellular volume fraction and protein content, underscoring the significance of protein–water interactions from the pathological point of view [17]. DSC-based approaches such as those employed in the present study may help to relate these observations to changes in distinct compartments of intracellular water in skeletal muscle in the future.

The observation that partially removing myosin increased the extra basal heat capacity suggests that rigor cross-bridge formation suppresses the heat sink capacity of actin. This interpretation is consistent with reports that actin–myosin interactions modify actin hydration properties [5] and implies that physiological dissociation of myosin heads during relaxation may enhance the thermal buffering function of actin-associated water.

At sub-zero temperatures, where bulk water is largely frozen, the persistence of extra basal heat capacity indicates the involvement of non-freezing water molecules. This observation is compatible with earlier reports of interfacial or hydration water populations associated with actin filaments [3,4] and suggests that such water contributes to muscle heat capacity, even when hydrogen bond rearrangements in bulk water are restricted at sub-zero temperatures.

To the best of our knowledge, no previous studies have directly linked the steady baseline heat capacity of muscles to actin-specific hydration effects under structurally defined sarcomeric conditions.

### 3.5. Limitations

Several limitations of the present study should be acknowledged.

First, the experiments were performed using skinned muscle fibers that contain multiple sarcomeric and associated proteins, making it difficult to attribute the observed thermal properties to a single protein species. Although actin and myosin constitute the vast majority of sarcomeric protein masses [14], regulatory proteins associated with thin filaments, such as troponin and tropomyosin, are not selectively manipulated and are partially removed together with actin during gelsolin treatment. Therefore, these regulatory proteins may have influenced the observed DSC results.

Second, we did not examine purified actin, myosin, or actomyosin systems. Future studies combining the DSC measurements of purified or reconstituted systems with those of structurally preserved muscle preparations will be important to further validate the present conclusions.

Third, frog skeletal muscles were used in this study. Although amphibian muscles have been widely employed in muscle physiology research, quantitative differences in filament lattice spacing, protein isoforms, and physiological temperature ranges may exist compared with mammalian muscles [7]. Accordingly, caution is required when extrapolating the absolute magnitude of heat capacity changes observed in mammalian or human skeletal muscles.

Finally, the experimental protocol involved cooling specimens to −80 °C. Although electron microscopy indicated that the untreated skinned muscle sarcomeric ultrastructure was largely preserved after freezing and subsequent heating to physiological temperatures, the subtle effects of deep freezing on protein–water interactions cannot be completely ruled out.

## 4. Materials and Methods

### 4.1. Specimens

The use of frogs (*Rana catesbeiana*) was approved by the Institutional Animal Care and Use Committee of the Jikei University (No. 2016-002) and conformed to the Guidelines for the Proper Conduct of Animal Experiments of the Science Council of Japan, issued in 2006. Sartorius muscle fibers from a frog were dissected and soaked for 1.5 h in relaxing solution (26.1 mM KMs, 5.7 mM MgMs_2_ (Ms, methanesulfonate, was used as a substitute for organic anions lost from the intracellular milieu, instead of chloride), 4.4 mM Na_2_ATP, 10 mM creatine phosphate, 10 mM EGTA [*O*,*O*’-Bis(2-aminoethyl)ethyleneglycol-*N*,*N*,*N*’,*N*’-tetraacetic acid], and 20 mM PIPES [Piperazine-1,4-bis(2-ethanesulfonic acid)] adjusted to pH 7.0 with KOH at 5 °C; estimated ionic strength 0.15) containing 0.5% triton X-100 to destroy the membrane system.

### 4.2. Electron Microscopy

For the electron microscopic observations, muscle specimens were prepared from the following experimental conditions: untreated skinned muscle before freezing and specimens subjected to DSC heating/cooling protocols from −80 °C to 30 °C, 60 °C, or 80 °C at a rate of 1 °Cmin^−1^.

For the electron microscopic observations, a specimen in 0.1 M phosphate buffer at 4 °C was fixed with 2% glutaraldehyde overnight and post fixed with 1% osmium tetroxide for 2 h. After dehydration in a graded ethanol series, specimens were placed in propylene oxide and embedded in Epok 812 (Okenshoji, Tokyo, Japan). Ultrathin sections were prepared using a diamond knife, stained with uranium acetate and lead citrate, and observed under an electron microscope (H-7500; Hitachi, Tokyo, Japan) at 80 kV.

### 4.3. Selective Removal of Myosin and Actin

For the removal of myosin from the thick filaments, a specimen was incubated in high-KCl solution (450 mM KCl, 5 mM MgCl_2_, 5 mM Na_2_ATP, 10 mM creatine phosphate, 10 mM EGTA, and 20 mM PIPES adjusted to pH 7.0 with KOH at 5 °C; ionic strength 0.45) for 60 min [18].

For the removal of actin on the thin filaments, a specimen was treated with gelsolin solution for 2 h in the absence of ATP (62.4 mM KMs, 1.5 mM MgMs_2_, 8.7 mM CaMs_2_, 10 mM EGTA, and 20 mM PIPES adjusted to pH 7.0 with KOH at 5 °C; ionic strength 0.15) and then incubated for 3 h with an additional 2 mM MgATP [19].

Electrophoresis was performed to evaluate myoprotein removal. The specimens were boiled in SDS solution at 95 °C for 6 min. After adding half the volume of glycerol bromophenol blue to the SDS sample solution, SDS-PAGE was performed on an 8% polyacrylamide gel. Protein molecular weight markers (Precision Plus Protein Dual Color Standards, Bio-Rad Laboratories, Hercules, CA, USA) were used to estimate the molecular weights. Detergent skinning rendered the sarcolemmal membrane permeable by dissolving membrane lipids, allowing the exchange of small soluble cytosolic components with the bathing solution while preserving the integrity of sarcomeric proteins within the fiber. Triton X-100 treatment largely reduces low–molecular-weight, freely diffusible soluble proteins [20], resulting in a protein composition of skinned muscle fibers that is dominated by abundant sarcomeric proteins such as myosin, actin and other regulatory myoproteins. To facilitate quantitative comparison of relative amounts of myosin heavy chain and actin, SDS samples were diluted to avoid signal saturation and allow reliable densitometric analysis of band intensities. Under these conditions, the major bands corresponding to myosin heavy chain and actin were readily identifiable based on their molecular weights and relative abundance. Protein molecular weight markers were included in representative gel runs to confirm the band identity. The density of Coomassie Brilliant Blue staining was read using a scanner (ChemiDoc Touch; Bio-Rad Laboratories) or a digital video camera (CMX-GH1; Sanyo, Osaka, Japan) and analyzed using ImageJ (version 1.51) [21].

### 4.4. Differential Scanning Calorimetry (DSC)

Prior to DSC measurements, a bundle of muscle fibers (approximately 30 fibers) was soaked in rigor solution (79.6 mM KMs, 1.6 mM MgMs_2_, 10 mM EGTA, and 20 mM PIPES adjusted to pH 7.0 with KOH at 5 °C; ionic strength 0.15) to deplete ATP within the fibers. They were then placed in a Tzero Aluminum Hermetic Pan (TA Instruments, New Castle, DE, USA) for the DSC measurements. Excess rigor solution around the muscle fibers was gently wiped with a piece of soft paper, and the pan seal was airtight.

DSC measurements were performed with a DSC Q2000 (TA instruments) at heating/cooling rates of 1 °Cmin^−1^, and an empty sealed pan was used as the reference to quantify the absolute heat flow and baseline heat capacity of the specimen, rather than the relative differences obtained by subtracting the solution or muscle containing reference traces. The actual thermal resistance between the heat source and the sensor was 27.3 KW^−1^ and between the sensor and the pan was 5.3 KW^−1^ when calibrated with Indium [22]. Cooling to −80 °C was required to ensure complete freezing of the aqueous component, allowing reliable quantification of ice-melting enthalpy and evaluation of steady baseline heat capacity over both sub-zero and physiological temperature ranges.

The measurements were performed from −80 °C with the following step-wise triple heating/cooling runs in succession: specifically, the specimens were subjected to three consecutive heating runs from −80 °C to 30 °C, followed by three runs from −80 °C to 60 °C (beyond the heat denaturation temperature of myosin), and finally three runs from −80 °C to 80 °C (beyond the denaturation temperature of actin). Because supercooling caused substantial variability in the cooling runs, only the heating runs (nine scans in total) were analyzed. Unlike conventional DSC analyses, in which the rescan baseline obtained after protein denaturation is subtracted, the present study analyzed raw heating scans without baseline subtraction. This approach was adopted to evaluate not only the discrete endothermic transitions but also the steady baseline level of the DSC curves, which reflects the apparent basal heat capacity of the muscle–solution system. This analysis was justified by the high baseline stability of the heat-flux DSC instrument, which allowed reliable comparison of steady baseline levels across different experimental conditions. For each heating run, the peaks were analyzed using TA Universal Analysis 2000 (TA Instruments). Peak curve fitting and baseline level evaluation were performed using MATLAB R2016a (MathWorks, Natick, MA, USA). After the measurement, the pan with the specimen was weighed to confirm the preserved mass of the sample at a 0.01 mg resolution (GH-202, A&D Company, Tokyo, Japan). Finally, the sample pan was unsealed and heated to 200 °C for 120 min to measure the evaporable content of the sample. The heat flow and specific heat capacity were expressed per evaporable weight (g).

### 4.5. Statistical Analysis

Experimental data are presented as the mean ± standard error of the mean (SEM). Prior to statistical analyses, data normality was assessed using the Shapiro–Wilk test and the homogeneity of variances was confirmed using Levene’s test. When these assumptions were satisfied, the representative properties deduced from the DSC measurements were statistically analyzed using Tukey’s post hoc test when significant differences were detected using ANOVA. The SDS-PAGE results were compared using a two-sample *t*-test, and all of the statistical analyses were performed using R (version 4.2.1; R Foundation for Statistical Computing, Vienna, Austria). Statistical significance was set at *p* < 0.05 for all analyses.

## 5. Conclusions

Using the DSC of skinned muscle fibers, we demonstrated that skeletal muscle exhibited a substantial extra basal heat capacity relative to the rigor solution alone in the physiological temperature range. Removal and denaturation experiments revealed that the extra basal heat capacity was primarily attributable to actin, whereas myosin played a modulatory role that partially suppressed the heat sink capacity of actin in the rigor state. The persistence of extra basal heat capacity even at sub-zero temperatures suggests the involvement of non-freezing water populations associated with actin filaments.

## Figures and Tables

**Table 1:** Position and area of 50 °C peak.

Muscle Type	Untreated (*n* = 7)	High-KCl-Treated (*n* = 27)			Gelsolin-Treated (*n* = 4)
		Sub-Peak A	Sub-Peak B	A + B	
Peak temperature [°C]	50.1 ± 0.3	44.1 ± 0.2	54.6 ± 0.1		48.8 ± 0.6
Peak area [J (g evaporable weight)^−1^]	0.938 ± 0.053	0.068 ± 0.005	0.089 ± 0.005	0.157 ± 0.006 **	0.846 ± 0.100

Expressed as mean ± SEM. ** *p* < 0.01 vs. untreated.

**Table 2:** Position and area of 67 °C peak.

Muscle Type	Untreated (*n* = 7)	High-KCl-Treated (*n* = 27)	Gelsolin-Treated (*n* = 4)
Peak temperature [°C]	67.2 ± 0.2	68.1 ± 0.1	66.8 ± 1.2
Peak area [J (g evaporable weight)^−1^]	0.283 ± 0.026	0.339 ± 0.019	0.085 ± 0.013 **

Expressed as mean ± SEM. ** *p* < 0.01 vs. untreated.

## Data Availability

The data that support the findings of this study are available within the article. Further inquiries can be directed to the corresponding author.
